# Implantable cardioverter-defibrillator use in patients with left ventricular assist device: prediction of ventricular arrhythmia using the VT-LVAD score

**DOI:** 10.3389/fcvm.2026.1707002

**Published:** 2026-02-26

**Authors:** Emmanuelle Massie, Jacinthe Boulet, Corrado De Marco, Pierre-Emmanuel Noly, Yoan Lamarche, Blandine Mondésert, Anique Ducharme

**Affiliations:** 1 Department of Medicine, Division of Cardiology, Montreal Heart Institute, Université de Montréal, Montreal, QC, Canada; 2 Department of Surgery, Montreal Heart Institute, Université de Montréal, Montreal, QC, Canada

**Keywords:** heart faiIure, heartfailure, ICD, LVAD (left ventricular assist device), ventricular arhythmias

## Abstract

**Background:**

The survival benefit of implantable cardioverter-defibrillators (ICD) in patients with left ventricular assist device (LVAD) remains unproven. The VT-LVAD score was developed to stratify arrhythmic risk and may help identify patients most likely to benefit from ICD therapy. We aimed to retrospectively assess its ability to identify patients at higher risk of ventricular arrythmias and to describe ICD-related complications in a population of patients with ICD and LVAD.

**Methods:**

A total of 63 primary continuous-flow LVAD implantation were performed at our institution between January 2010 and March 2020 were included and stratified by risk (VT-LVAD score <5 or ≥5). Thirty patients (47.6%) had a low/intermediate risk VT-LVAD score (<5) (Group 1) and 33 (52.4%) a high/very high-risk VT-LVAD (score ≥5) (Group 2). Patients either had a previous ICD or were implanted before discharge, unless transplanted urgently. Early postoperative outcomes, including in-hospital arrhythmic events with hemodynamic instability, were collected, along with long-term outcomes such as all-cause mortality, ICD therapies, and ICD-related complications.

**Results:**

Patients with a VT-LVAD score ≥5 were more likely to experience in-hospital ventricular arrhythmias (VAs) than those with score <5 (91% vs. 43%, *p* < 0.001). These VAs occurred mainly in the first five postoperative days, often due to an underlying cause, and resulted in hemodynamic instability in 40% of VT-LVAD <5 patients vs. 50% in VT-LVAD ≥5 (*p* = 0.44). Long-term mortality was similar for VT-LVAD <5 and ≥5 respectively (21.7% vs. 37.0%, *p* = 0.59) and there was no difference in arrhythmic events, including ATP therapies (17% vs. 22%, *p* = 0.73) and appropriate (0% vs. 4%) or inappropriate shocks (9% vs. 11%). There was one early lead dislodgement requiring repositioning, but no other long-term ICD complications.

**Conclusion:**

The findings of our study are exploratory and hypothesis-generating; while the VT-LVAD score identifies patients at higher early arrhythmic risk, long-term malignant VAs were rare in both groups, and no survival benefit of ICD therapy can be derived from this study.

## Introduction

Despite significant therapeutic advances over the last decade, heart failure (HF) remains the leading cause of hospital admission after the age of 65 and is associated with high morbidity and mortality, as well as substantial healthcare-related costs (1, 2). The overall survival of these patients decreases with disease severity and the cause of death varies according to symptom severity, with New York Heart Association (NYHA) functional class I–III patients dying more frequently from arrhythmic causes and NYHA class IV patients succumbing mostly to progressive pump failure (3, 4). Hence, current guidelines recommend an implantable cardioverter-defibrillator (ICD) in patients with heart failure with reduced ejection fraction (HFrEF) and NYHA class I–III (5). For highly selected patients with advanced HF and refractory symptoms, mechanical circulatory support with durable left ventricular assist devices (LVAD) has become an acceptable alternative; indeed, the most recent data with the HeartMate-3 LVAD demonstrated an overall survival close to 60% at 5 years (6). The current generation of continuous-flow LVAD (CF-LVAD) provides adequate cardiac output with lower occurrence of sudden cardiac death (SCD) and syncope, even in the presence of prolonged sustained ventricular arrhythmias (VAs) (7, 8).

The decision to implant (or re-activate) an ICD in LVAD patients is debatable, with many reports showing that VAs, including ventricular fibrillation (VF), is well tolerated in these patients (7, 8). There is currently much debate regarding whether implantation of an ICD reduces mortality in patients with CF-LVAD (9–13) and no randomized trial addressing this controversy has been performed to date. Yet, more than 80% of LVAD patients already have an ICD in place before LVAD implantation (14) and the International Society for Heart and Lung Transplantation (ISHLT) 2013 guidelines recommend reactivating ICDs in the postoperative period after LVAD implantation (Class I, LOE A) (15). For the minority of patients without an ICD prior to CF-LVAD implantation, the survival benefits of combining the two devices have not been demonstrated, with small case series and a meta-analysis from large registries reporting conflicting results (10–13, 16, 17).

Proponents for ICD implantation after LVAD rely on the high incidence of VAs, in particular in the early postoperative period, with reports of VAs in up to 35% of patients within 30 days (18), and its association with increased mortality, demonstrated at 54% vs. 18% in those without Vas (19). VAs in LVAD patients may lead to progressive myocardial dysfunction, with right-sided predominant hemodynamic consequences and intractable right ventricular (RV) failure, and/or thrombus formation secondary to myocardial standstill, all leading to reduced survival (7, 8, 20, 21). In contrast, ICDs may be associated with complications such as infection and bleeding (8), device interactions which were previously reported with earlier generation devices (22), all notwithstanding the potential psychological distress caused by shocks, whether appropriate or not (10). An alternative to transvenous ICDs (TV-ICD) to decrease the risk of infection, venous thrombosis, and lead failure is the use of subcutaneous ICDs (S-ICD) (23). Case reports have shown the absence of mechanical interference in patients with a S-ICD and implanted with either the HeartMate II or the Jarvik 2000 LVAD (23, 24). On the other hand, the absence of pacing and, especially, anti-tachycardia pacing (ATP) capabilities may be perceived as an obstacle to general adoption of this technology in CF-LVAD patients (25). To help clinicians with decision-making, a VT-LVAD score was recently proposed based on six independent predictive factors: VAs prior to LVAD implantation (2 points), no Angiotensin Converting Enzyme inhibitors (ACEI) post-LVAD (2 points), HF duration >12 months (2 points), VA post implantation <30 days (2 points), atrial fibrillation (AF) prior to LVAD (1 point), and idiopathic dilated cardiomyopathy (1 point) (26). This score has been validated in one single-center study and accurately predicted the occurrence of VAs in a cohort of 460 LVAD recipients (27).

Despite this limited evidence, our institution elected to implant all CF-LVAD patients with an ICD and to reactivate previously implanted ICD post-LVAD. Additionally, since 2015, we favored S-ICDs over TV-ICDs as the device of choice for new implantations post-LVAD in patients who do not require pacing nor ATP. In this context, we aimed to conduct a study assessing the incidence and clinical impact of sustained VAs following CF-LVAD implantation, and to evaluate whether the VT-LVAD score stratifies arrhythmic risk during the early postoperative period as well as during longer-term follow-up in an ICD-treated CF-LVAD population.

## Material and methods

### Study design

We performed a retrospective chart review of all patients aged 18 years and older implanted with a primary CF-LVAD (HeartMate-II and HeartMate-3) at the MHI between January 2010 and March 2020. As specified above, all these patients either had an ICD in place or were implanted with an ICD post-LVAD but prior to discharge; patients were excluded from the long-term outcomes analysis if they died or underwent cardiac transplantation during the index hospitalization prior to ICD implantation. Our primary objective was to assess the incidence and clinical impact of sustained ventricular arrhythmias after CF-LVAD implantation, while evaluating whether the VT-LVAD score stratifies arrhythmic risk during the early postoperative period and over longer-term follow-up. The secondary objectives included the incidence of ICD-related complications, such as inappropriate shock, device infection, and device-device interactions. Patients were stratified using the VT-LVAD score to assess the incidence of VAs and complications. Finally, as an exploratory analysis, we aimed to divide the CF-LVAD cohort between the two types of ICD, S-ICD and TV-ICD, to seek to propose a personalized approach for ICD in patients with CF-LVAD. This study was approved by our local institutional review and ethics boards.

Preoperative characteristics, catheterization reports, and echocardiography data were retrieved from the MHI mechanical circulatory support (MCS) electronic database and from the patients’ electronic medical records. Patients were retrospectively classified according to the VT-LVAD score described above as low/intermediate risk for VA if their score was <5 and high/very high risk for VA if their score was ≥5. All clinical follow-up and arrhythmic events were obtained from the MCS and cardiac transplantation and ICD databases, respectively. Baseline characteristics, in-hospital outcomes, and long-term outcomes were compared between the two groups. The occurrence of VAs was defined as sustained ventricular tachycardia (VT) or ventricular fibrillation (VF) of at least 30 s or terminated by ICD therapy. VAs were categorized as early if they occurred less than 30 days postoperatively and late if they happened 30 days or more postoperatively. The last follow-up was the first of either September 30th, 2021 or time of death or cardiac transplantation. Missing data were handled using complete-case analysis. The proportion of missing data was small and did not differ meaningfully between groups.

### Statistical analysis

Normality of continuous variables was assessed using the Kolmogorov–Smirnov test. Continuous variables are presented as mean ± standard deviation (SD) when normally distributed and as median with interquartile range (IQR) when non-normally distributed. Normally distributed continuous variables were compared between groups using the Student's *t*-test, whereas non-normally distributed continuous variables were compared using the Kruskal–Wallis test. Categorical variables are presented as frequencies and were compared using Fisher's exact test. All statistical analyses were performed using SPSS (IBM Corp., IBM SPSS Statistics for Mac, Version 22.0, Armonk, NY).

## Results

### Baseline characteristics

**Figure 1:**
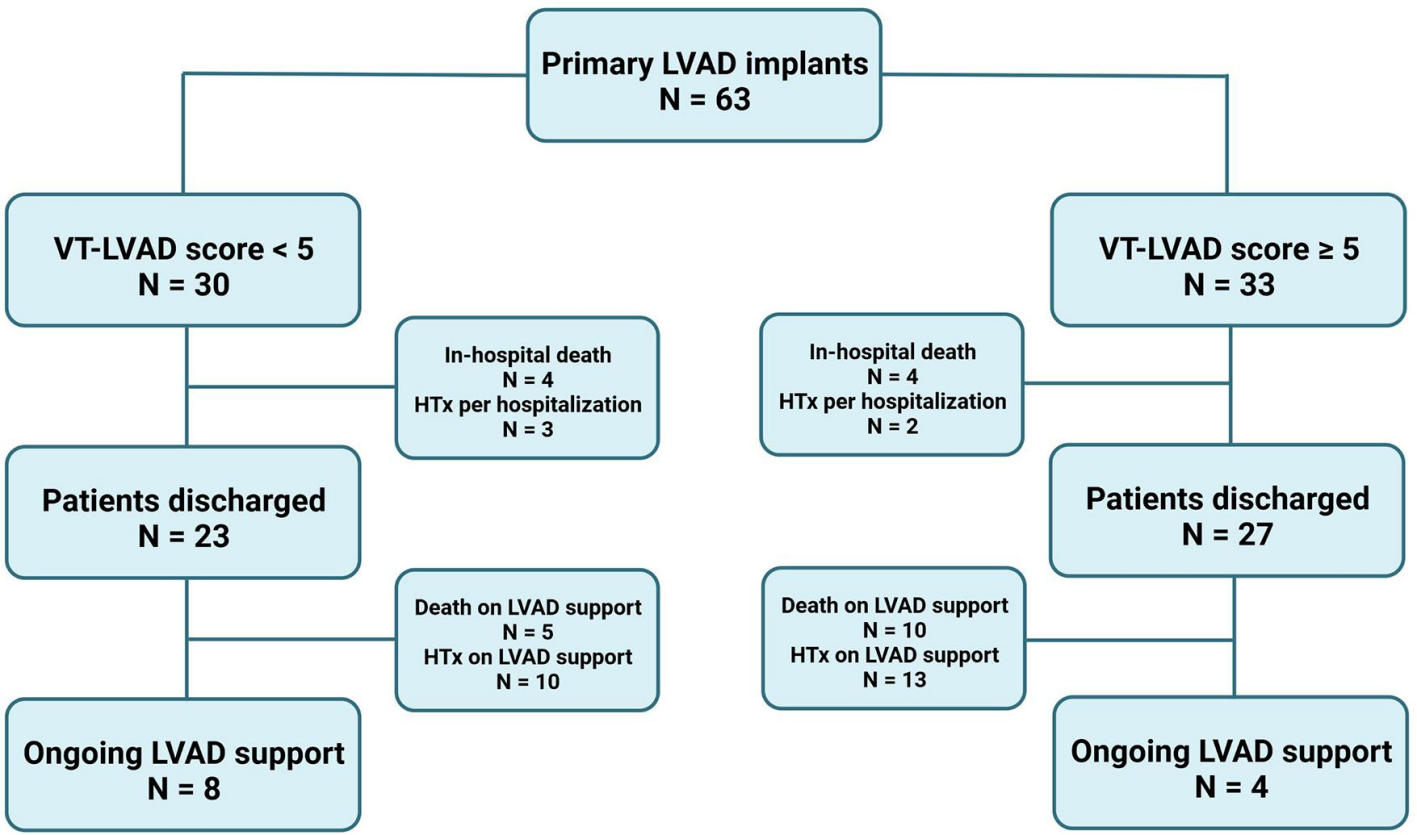
There were 4 in-hospital deaths in each group, and a total of 5 patients were transplanted before receiving an ICD (3 in the low/intermediate risk and 2 in the high/very high-risk group). A total of 15 patients died while on LVAD support (5 in the low/intermediate and 10 in the high/very high-risk group), and 23 (10 in the low/intermediate and 13 in the high/very high risk group) were transplanted during follow-up. At the end of the study, 8 patients in the low/intermediate and 4 in the high/very high-risk group were still under LVAD support.

Baseline characteristics are summarized in Table 1. A total of 63 primary CF-LVAD implantation were performed at the MHI during the study period, with a median (IQR) follow-up of 36 (59) months and a median duration of CF-LVAD support of 7.8 (20) months; 51 (81%) were implanted with HeartMate-II and 12 (19%) with HeartMate-3. Thirty patients (47.6%) had a low/intermediate risk VT-LVAD score (<5) (Group 1) and 33 (52.4%) a high/very high risk VT-LVAD (score ≥5) (Group 2). A consort chart describes the flow of the patients (Figure 1).

**Table 1:** Patients’ characteristics before LVAD implantation.

Patient characteristics	GROUP 1	GROUP 2	*p*-values
	VT LVAD score <5 (*n* = 30)	VT LVAD score ≥5 (*n* = 33)	
Age, years, median (IQR)	59 (12)	61 (12)	0.23
Sex, male	24 (80%)	27 (82%)	0.85
Ethnicity			
Caucasian	29 (97%)	28 (87%)	0.22
Black	1 (3%)	3 (9%)	
HF etiology			
Ischemic	15 (50%)	9 (28%)	0.56
Non-ischemic	8 (27%)	10 (30%)	
Other	2 (23%)	14 (42%)	
HF duration prior LVAD, months, median (IQR)	4 (113)	98 (123)	0.03
LVAD implant strategy			
Destination therapy	7 (23%)	8 (24%)	0.81
Bridge to transplantation	13 (43%)	14 (42%)	
Bridge to candidacy/decision	10 (33%)	10 (30%)	
INTERMACS classification			
1	4 (13%)	2 (6%)	0.24
2	9 (30%)	10 (30%)	
3	11 (37%)	7 (21%)	
4–5	6 (20%)	14 (42%)	
Temporary MCS prior to durable LVAD	5 (16%)	7 (21%)	
LVAD Type, HeartMate II	25 (83%)	26 (79%)	0.64
ICD implantation			
Before LVAD implantation	15 (50%)	27 (82%)	0.02
After LVAD implantation	9 (30%)	4 (12%)	
None	6 (21%)	2 (6%)	
Comorbidities			
Coronary artery disease	12 (40%)	11 (33)	0.58
Atrial fibrillation	5 (17%)	23 (70%)	0.001
CKD (eGFR < 60 mL/min/1.73 m^2^)	11 (37%)	19 (57%)	0.09
Diabetes	8 (27%)	13 (39%)	0.42
Hypertension	13 (43%)	15 (45%)	0.86
COPD	6 (21%)	9 (27%)	0.57
Arrhythmic history			
Prior VAs	10 (33%)	26 (79%)	0.02
Arrhythmic storm	2 (6%)	7 (21%)	0.34
ICD shocks prior to LVAD	5 (17%)	13 (39%)	0.35
Previous VA ablation	1 (3%)	4 (12%)	0.18
History of cardiac arrest	4 (13%)	10 (30%)	0.01
Medication			
Amiodarone,^a^ *n* (%)	7 (23%)	13 (39%)	0.19
Beta-blockers,^a^ *n* (%)	22 (73%)	30 (91%)	0.09
ACEI/ARA,^a^ *n* (%)	3 (10%)	9 (27%)	0.11
ARNI,^a^ *n* (%)	21 (70%)	22 (67%)	0.79
MRA,^a^ *n* (%)	17 (57%)	24 (73%)	0.20

CKD, chronic kidney disease; eGFR, estimated glomerular filtration rate; HF, heart failure; ICD, implantable cardioverter defibrillator; INTERMACS, Interagency Registry for Mechanically Assisted Circulatory Support; LVAD, left ventricular assist device; MCS, mechanical circulatory support; VAs, ventricular arrhythmias; ACE, angiotensin-converting enzyme inhibitor; ARA, angiotensin receptor antagonist; ARNI, angiotensin receptor neprilysin inhibitor; MRA, mineralocorticoid receptor antagonist.

^a^ Medication use at the time of LVAD implantation.

There was no difference between groups in age, sex, ethnicity, heart failure etiology, strategies at the time of CF-LVAD implantation, and Interagency Registry for Mechanically Assisted Circulatory Support (INTERMACS) profile. However, patients in Group 1 had shorter duration of HF, lesser history of atrial fibrillation (AF) (17% vs. 70% for Group 1 and 2 respectively, *p* = 0.001), fewer VAs prior to CF-LVAD implantation (33% and 79% for Group 1 and 2, *p* = 0.02), and lesser history of cardiac arrest (13% vs. 30% for Group 1 and 2, *p* = 0.01). There was also a trend toward less prior VA ablation (3% vs. 12% for Group 1 and 2, *p* = 0.18). Ultimately, less patients had an ICD in place at the time of CF-LVAD implantation (50% vs. 82% for Group 1 and 2, *p* = 0.02). In addition, patients in Group 1 were less likely to have a paced rhythm on the preoperative ECG (17% vs. 39% for Group 1 and 2, *p* = 0.01).

### In-hospital outcomes

In-hospital outcomes after CF-LVAD implantation according to the VT-LVAD score are presented in Figure 1 and Table 2. Early complications, in-hospital mortality and urgent cardiac transplantation were similar between the two groups, though with limited interpretability due to small numbers, except for one patient in Group 2 having RV lead dislodgement requiring re-intervention. However, there were significantly more patients in Group 2 experiencing VAs [30 (91%) compared to 13 (43%) in Group 1, *p* < 0.001]. Most of these VA episodes occurred in the first five postoperative days, in patients still receiving inotropic/vasopressor therapy, for both groups (4/5 in Group 1 and 10/14 in Group 2, *p* = 0.05) with a minority having electrolyte abnormalities, mainly hypomagnesemia (2/5 patients of Group 1 and 4/14 in Group 2, *p* = 0.74). Roughly 50% of sustained VA episodes in each group resulted in hemodynamic instability, defined by new or increasing vasopressor requirements (2/5 in Group 1 and 7/14 in Group 2, *p* = 0.44), without any difference in the rates of treated VAs between groups. One patient in each group developed early RV failure due to VAs.

**Table 2:** In-Hospital outcomes.

In-hospital outcomes	GROUP 1	GROUP 2	*p*-values
	VT LVAD score <5 (*n* = 30)	VT LVAD score ≥5 (*n* = 33)	
Length of stay post implant			
ICU [median (IQR)]	8 (11)	8 (12)	0.75
Total (median (IQR)	43 (7–90)	47 (21–68)	0.57
In-hospital mortality	5 (13%)	4 (12%)	0.60
Urgent cardiac transplantation	3 (10%)	2 (6%)	0.41
In-hospital complications			
Major infection	12 (40%)	12 (36%)	0.75
Stroke	3 (9%)	2 (6%)	0.75
Major bleeding	11 (36%)	16 (48%)	0.40
Acute kidney injury	14 (47%)	21 (64%)	0.22
Renal replacement therapy	7 (25%)	9 (27%)	0.84
RVF	7 (23%)	2 (6%)	0.32
RVF requiring MCS	4 (13%)	2 (6%)	0.55
Atrial fibrillation	11 (36%)	16 (48%)	0.16
Postoperative VA history			
Monitored VAs	13 (43%)	30 (91%)	<0.001
VAs treated			
Medication	3 (23%)	5 (17%)	0.68
ATP	2 (15%)	7 (23%)	0.69
Shock	1 (8%)	7 (23%)	0.39
RVF due to VAs	1 (3%)	1 (3%)	0.97

ICU, intensive care unit stay; LVAD, left ventricular assist device; RVF, right ventricular failure; MCS, mechanical circulatory support; HD, hemodynamic; VAs, ventricular arrhythmias.

### Long-term outcomes

**Figure 2:**
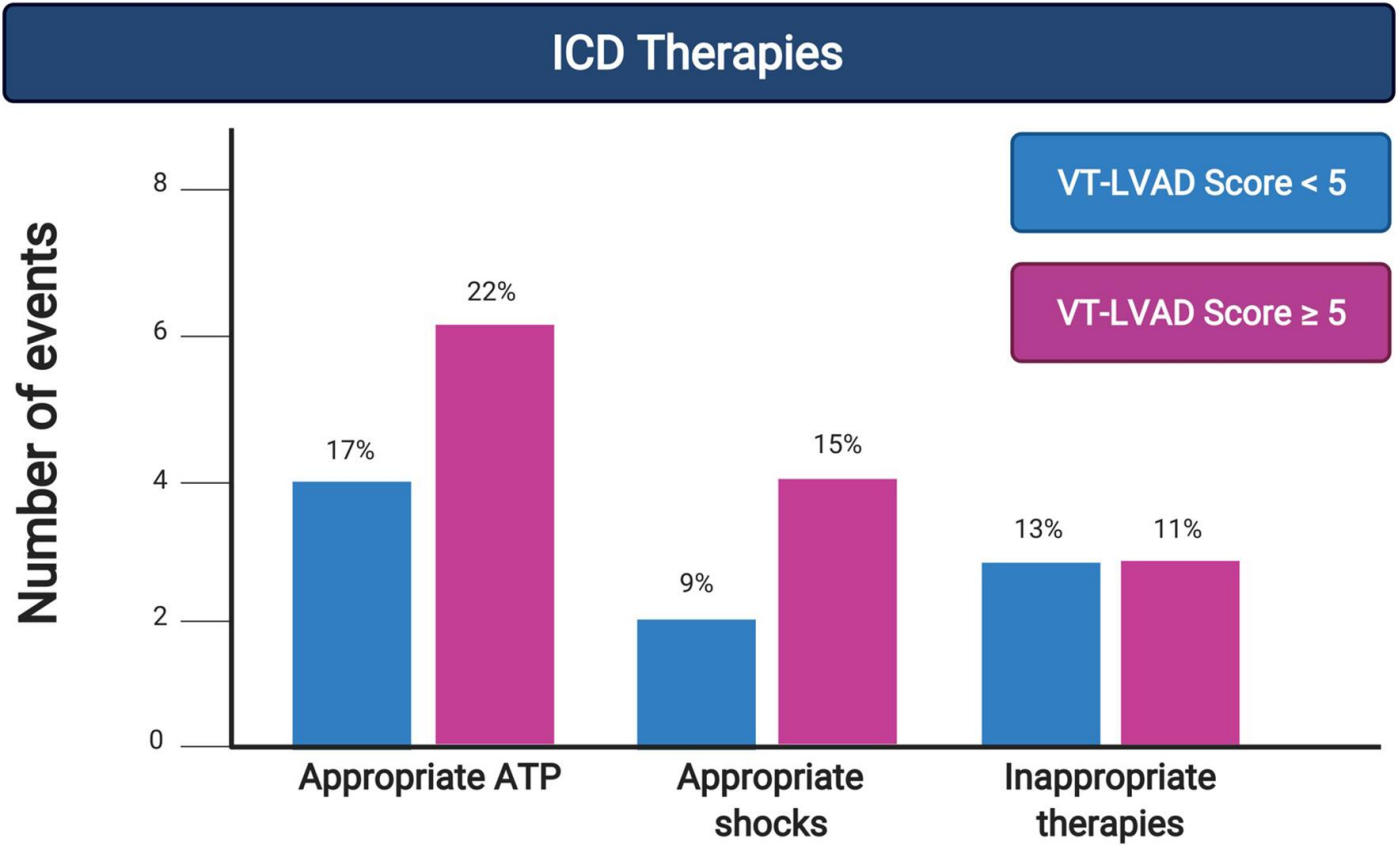
Long-term outcomes stratified by group as indicated (i.e., VT-LVAD Score <5 in blue, VT-LVAD Score ≥5 in grey) for outcomes of interest.

Long-term outcomes after hospital discharge are presented in Table 3 and Figure 2, with all percentages calculated using a denominator of patients discharged from their index hospitalization with a CF-LVAD in place. After a median follow-up of 50 (IQR 49) months, 15 patients died on LVAD support [5 (21.7%) in Group 1 and 10 (37.0%) in Group 2, *p* = 0.59], while 23 [10 (43.5%) in Group 1 and 13 (48.1%) in Group 2, *p* = 0.74] were transplanted. At the end of the study, 8 patients (34.8%) in Group 1 and 4 (14.9%) in Group 2 remained on LVAD support (*p* = 0.09). There was no difference in arrhythmic events between the two groups for appropriate therapies (ATP) (4 in Group 1 vs. 6 in Group 2). The same can be said about appropriate shocks (0 in Group 1 vs. 1 in Group 2) and inappropriate shocks (2 in Group 1 vs. 3 in Group 2); all inappropriate shocks were due to supra-ventricular tachycardia, and no formal statistical comparison were performed given the small numbers. When stratified by S-ICD vs. TV-ICD (Supplementary Table S2), there was no significant difference in device therapies, appropriate or inappropriate, between device types. There were no ICD-related infections, lead fractures, or other long-term ICD-related complications in the entire cohort.

**Table 3:** Long-term outcomes.

Long-term outcomes	GROUP 1	GROUP 2	*p*-values
	VT LVAD score <5 (*n* = 23)	VT LVAD score ≥5 (*n* = 27)	
Duration of support, months, median (IQR)	14 (55)	10 (16)	0.01
Heart transplantation	10 (43%)	13 (48%)	0.74
Death on LVAD support	5 (22%)	10 (37%)	0.59
ICD type			
Transvenous	18 (78%)	25 (93%)	0.22
Subcutaneous	5 (22%)	2 (7%)	0.15
ICD therapies			
ATP (nb of patients)	4 (17%)	6 (22%)	0.73
Any shocks (nb of patients)	2 (9%)	4 (15%)	0.67
Appropriate shocks (nb of patients)	0 (0%)	1 (4%)	0.42
Inappropriate shocks (nb of patients)	2 (9%)	3 (11%)	0.99
Competing mortality risk			
1 year	20%	21%	0.99
3 years	31%	32%	0.99

ATP, anti-tachycardia pacing; ICD, implantable cardioverter defibrillator; LVAD, left ventricular assist device; nb, number.

At the end of the follow-up period, 15 patients had died [5 (22%) in Group 1 vs. 10 (37%) in Group 2, *p* = 0.59]. Causes of death, in Groups 1 and 2 respectively, included VA refractory to anti-arrhythmic therapy [0 (0%) vs. 1 (7.7%), *p* = 0.19], terminal cardiomyopathy [2 (40%) vs. 3 (23%), *p* = 0.36], and LVAD-related complications such as pump thrombosis [1 (20%) vs. 3 (23%), *p* = 0.36], bleeding [0 (0%) vs. 1 (7.7%), *p* = 0.19], and neurological events [1 (20%) vs. 4 (30.8%), *p* = 0.24]. Other causes of death include lung cancer, pancreatitis, and complications post-kidney transplantation. Competing mortality risk was similar between groups at 1 year (20% and 21%, *p* = 0.99) and 3 years (31% and 32%, *p* = 0.99) for Group 1 and 2, respectively.

## Discussion

The role of implantable cardioverter-defibrillators (ICDs) in patients receiving continuous-flow left ventricular assist devices (CF-LVADs) remains controversial, and recent literature continues to challenge conventional wisdom regarding their utility and necessity. Although historically ICDs have been recommended in heart failure patients with significantly reduced ejection fractions to mitigate the risk of SCD, this benefit has not been clearly established in the LVAD-supported patient population.

Complementing these large registry-based analyses, single-center studies have similarly questioned the universal benefit of ICDs in LVAD patients. Data from the Cleveland Clinic highlighted substantial device-related complications, including infections and lead malfunctions, which introduce considerable morbidity risks. Such observations emphasize the critical need for a careful risk-benefit evaluation when contemplating ICD implantation. These findings highlight a broader clinical perspective: the potential adverse effects associated with device implantation may outweigh the theoretical benefits in patients supported by CF-LVADs.

The VT-LVAD score, initially developed by Galand et al., represents an important advancement in clinical risk stratification, providing clinicians with a structured approach to evaluating the individual risk of ventricular arrhythmias in CF-LVAD-supported patients. Recent external validations of this scoring system have reinforced its predictive accuracy and clinical relevance. For instance, a multicenter validation study by Darma et al. demonstrated that patients identified as high-risk by the VT-LVAD score indeed experienced significantly higher rates of late ventricular arrhythmias compared to lower-risk counterparts. This consistency across studies underscores the utility of the VT-LVAD score in stratifying risk and potentially guiding clinical decision-making regarding ICD implantation and follow-up strategies.

In the present study, we similarly observed that a higher VT-LVAD score effectively identified patients at increased risk of early postoperative VAs. However, our analysis did not show a significant difference in long-term mortality or rates of late arrhythmic events between high-risk and low-risk groups. This absence of difference in long-term outcomes despite clear differences in early postoperative arrhythmic events aligns with recent literature and highlights the complexity of clinical outcomes in this population. Specifically, these findings suggest that while the VT-LVAD score is highly predictive of early postoperative arrhythmic complications, its predictive power regarding long-term outcomes remains uncertain. Such observations underscore the need for further refinement of predictive tools to enhance their utility in long-term clinical decision-making.

Moreover, the choice between different ICD modalities, namely transvenous ICDs (TV-ICDs) and subcutaneous ICDs (S-ICDs), adds another layer of complexity. While TV-ICDs provide the advantage of anti-tachycardia pacing, a crucial function for patients prone to recurrent monomorphic ventricular tachycardia, they are associated with increased risks of infections and lead-related complications. Conversely, S-ICDs mitigate many of these risks due to their entirely subcutaneous placement, yet their inability to deliver pacing therapies may limit their use in certain LVAD-supported patients. Recent observational studies and case series have begun exploring the efficacy and safety of S-ICDs in this unique population. Preliminary data have suggested minimal electromagnetic interference with CF-LVADs, indicating S-ICDs could offer a viable alternative for patients at lower risk of recurrent monomorphic arrhythmias who do not require pacing support.

Despite these insights, our current understanding remains incomplete, and significant gaps in evidence persist. One major limitation, as underscored by this and other studies, is the retrospective nature and single-center designs, limiting generalizability and conferring susceptibility to unmeasured confounding. Indeed, the absence of randomized controlled trials directly addressing ICD use in CF-LVAD patients remains a critical limitation in current clinical guidelines and decision-making processes. Moreover, as CF-LVAD technology continues to evolve, particularly with newer-generation devices demonstrating improved hemodynamic profiles and potentially altered arrhythmic risks, older data may rapidly become outdated. Therefore, contemporary randomized trials evaluating ICD utility in CF-LVAD patients, stratified by risk (such as VT-LVAD score), are critically needed to provide definitive guidance.

Future studies should also explore the psychological impact of ICD shocks in CF-LVAD patients, given the documented adverse effects of inappropriate and appropriate ICD therapies on quality of life. The psychosocial burden and potential impact on patient adherence and clinical follow-up underscore the need for comprehensive patient-centered approaches. It is paramount for clinicians to incorporate patient preferences and quality-of-life considerations into decision-making frameworks, alongside clinical risk stratification tools such as the VT-LVAD score.

We reported the incidence of VAs and ICD complications in all patients with primary CF-LVAD implantation at our center between 2010 and 2020, stratified according to their arrhythmic risk using the VT-LVAD score. Salient findings are: (1) our cohort was almost equally divided between low/intermediate risk (Group 1, score <5) and high/very high risk (Group 2, score ≥5); (2) patients with higher VT-LVAD scores experienced significantly more early postoperative arrhythmic events than their low/intermediate risk counterparts; (3) there was a high number of long-term VAs in both groups, with no significant difference between groups; (4) while the VT-LVAD score was strongly associated with early postoperative VAs, differences in long-term arrhythmic outcomes were not statistically significant, a finding that may be influenced by limited sample size and power.

There is no available data from randomized-controlled trials to help the clinician decide whether to implant an ICD in primary prevention for patients with a CF-LVAD. Only a few studies have evaluated the potential survival benefit of ICD therapy in CF-LVAD patients, with conflicting results (9, 12, 18, 28). We showed that a higher VT-LVAD score was associated with a higher risk of early VAs and a trend toward more late VAs and ICD therapies, but we could not show any impact on mortality or urgent cardiac transplantation listing. Nonetheless, this subgroup of patients may still benefit from ICD implantation after CF-LVAD. While the VT-LVAD score was developed by Galand et al. for patients implanted mostly with previous generation CF-LVADs (HeartMate-II, HeartWare, and Jarvik 2000), it has been validated by Darma et al. in their cohort of LVAD patients (HeartWare: 50%; HeartMate-3: 42%). Indeed, they showed that a score of 5 and more was an independent predictor of late VAs (odds ratio 4.8; *p* < 0.001) (27). Our results are concordant with their findings and suggest that the VT-LVAD score can also be applied to the HeartMate-3 population, the only available durable LVAD on the market today. Unfortunately, whether there was a survival benefit associated with ICD implantation in high-risk patients cannot be derived from current studies, ours included.

Nevertheless, the vast majority of our patients (82%) in the high/very high-risk group already had an ICD before CF-LVAD implantation and had previous episodes of VAs (79%), compared to 50% with pre-LVAD ICD in the low/intermediate risk group. This is not surprising given patients in the latter group had shorter disease duration, less VAs, and less comorbidities, which represent elements included in the score. Despite these findings, INTERMACs scores were not statistically different between the two groups.

Improving advanced HF patient care by assessing more accurately their risk of SCD is important as it may enable clinicians to avoid the cost and potential risks of ICD-related complications. ICD-associated infections have been reported to be as high as 7% (29), and outcomes of patients with infections and concomitant LVAD and ICD are poor (30). Moreover, patients undergoing ICD therapies have been shown to suffer from decreased quality of life (31, 32). Of note, one patient received an inappropriate shock during the index hospitalization due to electromagnetic interference upon reactivation of their S-ICD post-LVAD implantation. The problem was rectified by changing the programmed shock vector on the S-ICD in question. No other early inappropriate shocks occurred. During long-term follow-up, a significant number (9% and 11% for Group 1 and 2 respectively) of inappropriate shocks due to supraventricular tachyarrhythmias were noted. These results are similar to the reported 7.3% incidence of inappropriate shocks in CF-LVAD patients by Dharmavaram et al. (33). The presence of such potential risks supports a personalized approach to SCD risk assessment and management in the CF-LVAD population.

The optimal type of ICD, TV-ICD or S-ICD, to combine with a CF-LVAD has yet to be determined. Although we only had seven S-ICD in our cohort, a few studies suggest that the S-ICD could represent an interesting alternative to the TV-ICD in LVAD-assisted patients or in patients with advanced HF awaiting transplantation (34, 35). While evidence for the use of the S-ICD as a way to decrease lead-related complications in patients not requiring pacing is mounting (36, 37), the S-ICD's benefit remains uncertain in CF-LVAD patients and, more specifically, in patients with previous monomorphic VT. Moreover, there are notable limitations associated with the S-ICD, particularly inability for pacing, including anti-tachycardia pacing, and possible interactions between the S-ICD and the CF-LVAD because of electromagnetic interference.

## Limitations

There were noteworthy limitations in our study. This was a small single-center retrospective study, which gives little power to our interpretation. Our results may have been influenced by confounding factors that we did not account for due to the observational nature of our study design. Owing to the small cohort size and high collinearity between key clinical variables and elements of the VT-LVAD score, multivariable adjustment was not performed, as it would have risked model instability and overfitting. The results should therefore be interpreted cautiously and considered exploratory and hypothesis-generating.

Every patient at our center was implanted with an ICD prior to hospital discharge, except for those transplanted or deceased during the index hospitalization, thus it was not possible for us to compare the outcomes of CF-LVAD patients with or without ICD. In addition, there was no standardization in ICD programming for the early patients in our cohort and a few patients had their ICD follow-up at other institutions. Reports from ICD interrogations were sent to us retrospectively, but some information may have been missed with respect to VAs. Lastly, we did not compute an area under the curve which would have been helpful, as the VT-LVAD score was not re-derived or recalibrated in this cohort and the number of outcome events was insufficient to support robust discrimination analyses.

## Conclusion

Our retrospective analysis showed that the VT-LVAD score effectively identified patients at higher risk of early postoperative ventricular arrhythmias. However, late malignant arrhythmias were uncommon in both risk groups, and no mortality difference related to ICD therapy could be demonstrated. These observations must be interpreted cautiously given the study's limitations, including the small, single-center cohort and the absence of a comparator group, as all patients received an ICD. Notably, ICD-related complications in our center were infrequent, and inappropriate shock rates were similar across risk groups. This highlights the need to balance the potential risks and benefits of routine ICD implantation in LVAD-supported patients, particularly beyond the early postoperative phase.

Overall, the VT-LVAD score may help identify patients who are more likely to experience early arrhythmic events, but whether this translates into a meaningful long-term benefit from ICD therapy remains unknown. As this study is exploratory and hypothesis-generating, prospective randomized trials are required to determine the true clinical value of ICD implantation in LVAD recipients stratified by arrhythmic risk.

## Data Availability

The raw data supporting the conclusions of this article will be made available by the authors, without undue reservation.
