# Supplementary material for: Implantable cardioverter-defibrillator use in patients with left ventricular assist device: prediction of ventricular arrhythmia using the VT-LVAD score
Source: Front Cardiovasc Med. 2026 Feb 26;13:1707002. doi: 10.3389/fcvm.2026.1707002 (PMC12979384; doi:10.3389/fcvm.2026.1707002)
Supplement: Supplementary file 1 [file Datasheet1.docx]

**Supplementary Material**

**Table S1: Pre-Operative Electrocardiographic, Echocardiographic, Hemodynamic, and Laboratory Data**

|  | **GROUP 1**  **VT LVAD Score <5**  ***(n=30*)** | **GROUP 2**  **VT LVAD Score ≥5**  **(*n=*33)** | ***p*-values** |
| --- | --- | --- | --- |
| Pre-Operative ECG |  |  |  |
| Sinus Rhythm | 23 (77%) | 18 (55%) | 0.11 |
| QT Interval >450ms | 18 (60%) | 21 (64%) | 0.14 |
| Atrial Fibrillation | 6 (6%) | 2 (6%) | 0.97 |
| QRS >120ms | 20 (67%) | 17 (51%) | 0.62 |
| Paced Rhythm | 5 (17%) | 13 (39%) | 0.01 |
| Echocardiographic Parameters |  |  |  |
| LVEF (%) | 19±7 | 19±6 | 0.46 |
| LVEDD (mm) | 72±10 | 67±10 | 0.79 |
| RV Function (qualitative) |  |  |  |
| Normal | 9 (33%) | 9 (25%) | 0.57 |
| Mild Dysfunction | 9 (33%) | 13 (36%) | 0.99 |
| Moderate Dysfunction | 10 (37%) | 14 (39%) | 0.69 |
| Hemodynamic Data (mean±SD) |  |  |  |
| Systolic Blood Pressure (mmHg) | 93±16 | 93±14 | 0.97 |
| Mean PA Pressure (mmHg) | 34±10 | 36±9 | 0.56 |
| Mean RA Pressure (mmHg) | 10±5 | 11±7 | 0.69 |
| Mean PCWP (mmHg) | 24±8 | 25±7 | 0.50 |
| PAPI | 5.0±4.9 | 3.3±1.7 | 0.15 |
| PVR (Wood Units) | 3.1±1.5 | 3.0±1.9 | 0.94 |
| RVSWI (g/m/beat/m^2^) | 9.0±4.5 | 8.7±3.5 | 0.81 |
| Cardiac Index (L/min/m^2^) | 2.1±0.7 | 1.9±0.6 | 0.33 |
| Laboratory Parameters, mean ± SD |  |  |  |
| Hemoglobin (g/L) | 108±23 | 104±18 | 0.24 |
| Creatinine (umol/L) | 136±49 | 170±60 | 0.02 |
| Glomerular filtration rate, ml/min/1.73m2 | 53±38 | 45±21 | 0.32 |
| BUN (mmol/L) | 16±10 | 18±10 | 0.77 |
| Bilirubin (umol/L) | 25±14 | 29±27 | 0.18 |
| Sodium (mEq/L) | 132±6 | 131±4 | 0.32 |
| NT-proBNP (pg/L) | 9494±6743 | 9817±7767 | 0.68 |

BUN: blood urea nitrogen, ECG: electrocardiogram, LVEDD: left ventricular end-diastolic diameter, LVEF: left ventricular ejection fraction, NT-proBNP: N-terminal pro-brain natriuretic peptide, PA = pulmonary artery; RA = right atrium; PCWP = pulmonary capillary wedge pressure; PAPI = pulmonary artery pulsatility index; PVR = pulmonary vascular resistance; RVSWI = right ventricular stroke work index

**Supplementary Table S2: VT-ICD vs S-ICD**

|  | **TV-ICD (*n*=43)** | **S-ICD (*n*=7)** |
| --- | --- | --- |
| ATP (nb patients) | 10 | N/A |
| Any Shocks (nb patients) | 2 | 4 |
| Appropriate Shocks (nb patients) | 1 | 0 |
| Inappropriate Shocks (nb patients) | 4 | 1 |
